# Incidence, Clinical Outcomes, and Factors Associated With Post-intubation Hypotension Among Critically Ill Patients Undergoing Emergency Endotracheal Intubation: A Retrospective Observational Study

**DOI:** 10.7759/cureus.115316

**Published:** 2026-08-27

**Authors:** Hafiza Amber Noreen Fatima, Shahryar Shaukat, Rohma Qureshi, Savaid Ali, Amna Rehman, Saud Gul, Hamza Tahir, Hafiz Umair Zulfiqar Ali

**Affiliations:** 1 Emergency Medicine, Watford General Hospital, Watford, GBR; 2 Psychiatry, Letterkenny University Hospital, Letterkenny, IRL; 3 Internal Medicine, Shalamar Hospital, Lahore, PAK; 4 Internal Medicine, Lady Reading Hospital, Peshawar, PAK; 5 Internal Medicine, Islamabad Medical and Dental College, Islamabad, PAK; 6 Internal Medicine, Royal Derby Hospital, Derby, GBR; 7 Internal Medicine, Lahore Medical and Dental College, Lahore, PAK

**Keywords:** airway, apache-ii score, hypotension, post-intubation, sofa score

## Abstract

Background

Post-intubation hypotension (PIH) is a common and clinically significant complication of emergency airway management in critically ill patients and is associated with adverse short-term outcomes.

Objective

The objective of this study was to determine the incidence, clinical outcomes, and factors associated with post-intubation hypotension among critically ill adults undergoing emergency endotracheal intubation.

Methodology

This retrospective observational study was conducted at Mayo Hospital, Lahore, Pakistan. Medical records of 234 adult patients who underwent emergency endotracheal intubation between March 2023 to March 2026 were reviewed. PIH was defined as systolic blood pressure <90 mmHg, mean arterial pressure <65 mmHg, or a ≥20% reduction in systolic blood pressure from the pre-intubation baseline within 30 minutes after successful intubation. Multivariable logistic regression was performed to identify factors independently associated with PIH.

Results

PIH occurred in 68 of 234 patients (29.1%). Patients with PIH were significantly older and had higher Acute Physiology and Chronic Health Evaluation II (APACHE II), Sequential Organ Failure Assessment (SOFA) score, and Shock Index values than those without PIH. On multivariable analysis, increasing age (adjusted OR (AOR) 1.03, 95% CI 1.01-1.06; p = 0.011), chronic kidney disease (AOR 2.08, 95% CI 1.01-4.29; p = 0.047), sepsis (AOR 2.64, 95% CI 1.38-5.05; p = 0.003), higher APACHE II score (AOR 1.11, 95% CI 1.04-1.18; p = 0.001), elevated Shock Index (AOR 3.42, 95% CI 1.74-6.74; p < 0.001), ketamine use compared with etomidate (AOR 1.89, 95% CI 1.01-3.53; p = 0.045), and difficult airway (AOR 2.27, 95% CI 1.07-4.83; p = 0.033) were independently associated with PIH. PIH was also associated with higher vasopressor requirement, longer mechanical ventilation and ICU and hospital stays, increased AKI, and higher ICU and in-hospital mortality.

Conclusion

PIH occurred in nearly one-third of critically ill adults undergoing emergency endotracheal intubation and was associated with worse short-term clinical outcomes. Older age, sepsis, chronic kidney disease, higher APACHE II score, elevated Shock Index, ketamine use, and difficult airway were independently associated with PIH. These findings support early recognition of patients at increased risk and warrant prospective multicentre studies to determine whether standardized peri-intubation strategies can reduce PIH and improve outcomes.

## Introduction

Post-intubation hypotension (PIH) is one of the most common and clinically significant complications following emergency endotracheal intubation in critically ill patients, occurring shortly after airway establishment and frequently leading to hemodynamic deterioration [1]. It is commonly defined as a reduction in systolic blood pressure (SBP) to <90 mmHg, a mean arterial pressure (MAP) <65 mmHg, or a clinically significant decrease in blood pressure within the first 30 minutes after successful endotracheal intubation, although definitions vary across studies [2]. Emergency endotracheal intubation is a life-saving intervention performed in critically ill patients with respiratory failure, shock, severe trauma, altered mental status, and other life-threatening conditions requiring immediate airway protection. However, the transition from spontaneous to positive-pressure ventilation, combined with the cardiovascular effects of induction agents and airway manipulation, can precipitate acute hemodynamic instability, particularly in patients with limited physiological reserve [3].

Patients requiring emergency intubation often have underlying conditions such as hypovolemia, septic shock, cardiogenic shock, metabolic acidosis, severe hypoxemia, or major trauma, all of which increase susceptibility to cardiovascular collapse during induction of anesthesia and initiation of mechanical ventilation [4]. Positive-pressure ventilation reduces venous return and cardiac output, while induction agents may further decrease myocardial contractility and systemic vascular resistance, thereby increasing the risk of post-intubation hypotension. Consequently, PIH has emerged as an important quality indicator of emergency airway management because of its association with preventable adverse clinical outcomes [5].

Emergency endotracheal intubation is one of the most frequently performed life-saving procedures in emergency departments (EDs) and intensive care units (ICUs) worldwide [6]. Previous studies have reported that PIH occurs in approximately 20-40% of critically ill adults undergoing emergency airway management, although the reported incidence varies according to patient characteristics, illness severity, and the definition of hypotension used [7]. PIH has consistently been associated with adverse outcomes, including increased vasopressor requirements, prolonged mechanical ventilation, longer ICU and hospital stays, acute kidney injury (AKI), and higher mortality. Despite its clinical importance, evidence regarding the incidence and predictors of PIH from low- and middle-income countries, including Pakistan, remains limited, where emergency intubations are frequently performed for sepsis, trauma, poisoning, respiratory failure, neurological emergencies, and cardiovascular disorders [8].

The development of PIH is multifactorial and reflects the interaction between patient-related, disease-related, and procedural factors [9]. Patient characteristics such as advanced age, pre-existing hypotension, sepsis, chronic kidney disease, heart failure, hypovolemia, metabolic acidosis, elevated lactate levels, and greater illness severity have all been associated with an increased risk of PIH [10]. Procedural factors, including multiple intubation attempts, prolonged laryngoscopy, difficult airway, inadequate preoxygenation, and the choice of induction agent, may further contribute to peri-intubation hemodynamic instability [11,12]. Although etomidate is generally considered to have greater hemodynamic stability than other induction agents, the effects of ketamine remain controversial, particularly in critically ill patients with catecholamine depletion, and the optimal induction strategy continues to be debated [13,14]. Early identification of patients at increased risk for PIH may facilitate targeted preventive measures, including optimization of hemodynamic status, careful selection of induction agents, and timely vasopressor support, thereby improving peri-intubation safety and clinical outcomes.

Objective

The primary objective of the study was to determine the incidence of PIH among critically ill adults undergoing emergency endotracheal intubation in a single-center emergency department. The secondary outcomes were identification of factors independently associated with PIH and evaluation of its association with vasopressor requirement, duration of mechanical ventilation, ICU length of stay, hospital length of stay, AKI, ICU mortality, and in-hospital mortality.

## Materials and methods

This was a retrospective observational study conducted at the Emergency Department of Mayo Hospital, Lahore, Pakistan, using medical records of critically ill adults who underwent emergency endotracheal intubation between March 2023 and March 2026. Hospital electronic medical records and patient charts were reviewed to identify eligible patients.

Study population

Inclusion and Exclusion Criteria

Patients aged ≥18 years who underwent emergency endotracheal intubation in the emergency department during the study period and had complete documentation of PIH parameters, intubation characteristics, and post-intubation clinical outcomes were eligible for inclusion. Patients were eligible irrespective of the underlying critical illness or indication for intubation, including those with cardiovascular diseases.

Patients younger than 18 years, who underwent elective rather than emergency intubation, were intubated before arrival at the emergency department, had repeated intubations during the same hospital admission, were receiving vasopressor therapy before intubation, received palliative or comfort-focused care, or had incomplete medical records were excluded.

Sample Size

**Figure 1:**
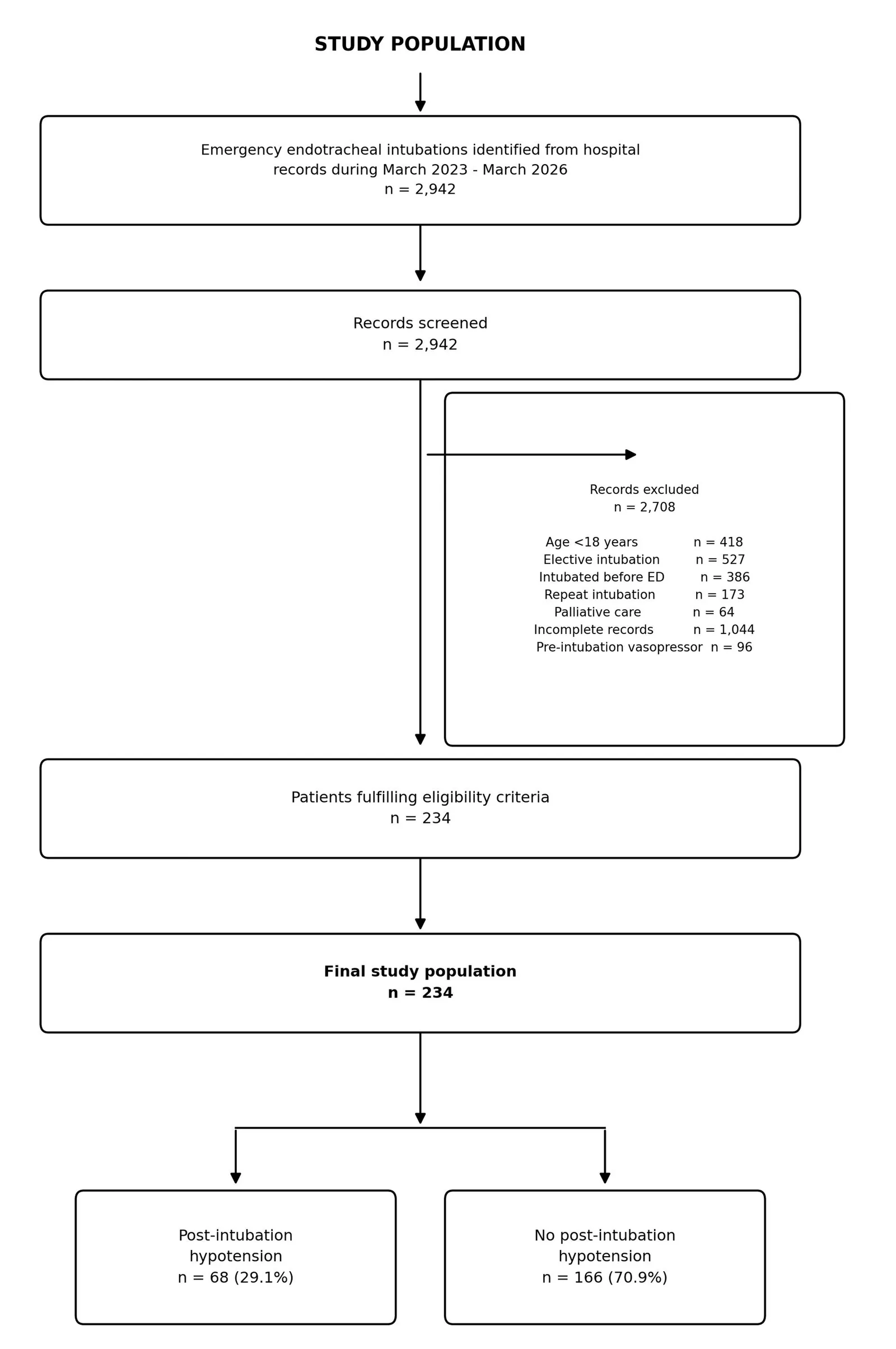
STROBE flow diagram of patient selection STROBE: STrengthening the Reporting of OBservational studies in Epidemiology

A non-probability consecutive sampling technique was employed. During the study period, a total of 2,942 emergency endotracheal intubation records were initially identified from hospital records. After screening, 2,708 records were excluded according to predefined exclusion criteria. A total of 234 patients fulfilled all eligibility criteria and were included in the final analysis. The participant selection process is illustrated in the STROBE (STrengthening the Reporting of OBservational studies in Epidemiology) flow diagram (Figure 1).

Definitions

Post-Intubation Hypotension

PIH is defined as the occurrence of any of the following within 30 minutes after successful endotracheal intubation: SBP <90 mmHg, MAP <65 mmHg, or a reduction of ≥20% in SBP from the pre-intubation baseline. The lowest blood pressure recorded during the first 30 minutes following intubation was used for outcome assessment.

Pre-intubation blood pressure was recorded immediately before induction and served as the baseline hemodynamic measurement. Patients were not excluded solely on the basis of PIH, provided they met the other eligibility criteria. PIH was determined exclusively from blood pressure changes occurring during the 30-minute period after successful intubation.

Critically Ill

For this study, “critically ill” refers to patients requiring emergency endotracheal intubation because of acute or potentially life-threatening physiological or clinical deterioration requiring airway and ventilatory support. The underlying indication for intubation was recorded, and no specific disease category was excluded solely on the basis of diagnosis.

Difficult Airway

A difficult airway was defined as an intubation in which the treating clinician documented difficulty with endotracheal tube placement and/or three or more intubation attempts were required to achieve successful tracheal intubation.

Data collection

Data were extracted retrospectively from electronic medical records and patient charts using a standardized data collection proforma. Demographic characteristics including age, sex, body mass index (BMI), and pre-existing comorbidities were recorded. Clinical variables included the indication for intubation, pre-intubation systolic blood pressure, heart rate, Shock Index (SI) [15], Sequential Organ Failure Assessment (SOFA) score [16], Acute Physiology and Chronic Health Evaluation II (APACHE II) score [17], induction agent used, first-pass intubation success, number of intubation attempts, difficult airway, and peri-intubation complications.

APACHE II and SOFA scores were recorded as baseline measures of illness severity using the clinical and laboratory information available before endotracheal intubation. Post-intubation physiological measurements were not used as baseline predictors in the regression analysis.

Clinical outcomes recorded included PIH, vasopressor requirement after intubation, duration of mechanical ventilation, ICU length of stay, hospital length of stay, AKI, ICU mortality, and in-hospital mortality.

Vasopressor requirement was assessed as new initiation of vasopressor therapy after intubation; patients receiving vasopressors before intubation were excluded. AKI was assessed as a post-intubation clinical outcome based on the available renal function data documented after intubation.

The induction agents used for emergency endotracheal intubation were recorded from the medical and airway procedure records. In the present cohort, ketamine and etomidate were the only induction agents used, and each patient received one of these two agents before intubation. No patients were documented as receiving propofol, midazolam, or other induction agents, and no eligible intubations were performed without an induction agent.

Data analysis

Data were analyzed using IBM SPSS Statistics for Windows, version 26.0 (IBM Corp., Armonk, New York, United States). Continuous variables were assessed for normality using the Shapiro-Wilk test and are presented as mean ± standard deviation (SD). Categorical variables are presented as frequencies and percentages. Comparisons between patients with and without PIH were performed using the independent-samples t-test for continuous variables and the Pearson chi-square test or Fisher's exact test for categorical variables, as appropriate. Corresponding test statistics and two-sided p-values were reported.

Variables with a p-value <0.20 in univariable analysis or considered clinically relevant based on previous literature were entered into a multivariable binary logistic regression model to identify factors independently associated with PIH. Regression results are presented as β coefficients, standard errors (SEs), Wald χ² statistics, adjusted odds ratios (AORs), 95% confidence intervals (CIs), and p-values. Multicollinearity among independent variables was assessed before model construction, and variables demonstrating significant collinearity were not entered simultaneously into the final model.

Model performance was assessed using the omnibus test of model coefficients, Hosmer-Lemeshow goodness-of-fit test, Cox and Snell R², and Nagelkerke R². Separate multivariable logistic regression models were constructed to evaluate the association between PIH and selected adverse clinical outcomes after adjustment for clinically relevant baseline covariates. A two-sided p-value <0.05 was considered statistically significant.

## Results

A total of 234 critically ill patients who underwent emergency endotracheal intubation were included in the analysis. Of these, 68 patients (29.1%) developed PIH, whereas 166 patients (70.9%) remained hemodynamically stable following intubation.

Patients who developed PIH were significantly older than those who did not (63.8 ± 14.2 vs. 56.1 ± 15.1 years; t = 3.44, p = 0.001). Mean BMI was slightly higher in the PIH group (28.2 ± 4.4 vs. 27.1 ± 4.1 kg/m²; t = 1.74, p = 0.083), while the proportion of male patients was comparable between the two groups (n=41, 60.3% vs. n=98, 59.0%; χ² = 0.03, p = 0.853). Chronic kidney disease (n=18, 26.5% vs. n=23, 13.9%; χ² = 5.09, p = 0.024) and sepsis (n=35, 51.5% vs. n=46, 27.7%; χ² = 11.86, p < 0.001) were significantly more common among patients who developed PIH, whereas the prevalence of trauma did not differ significantly between the groups (n=12, 17.6% vs. n=42, 25.3%; χ² = 1.60, p = 0.206). Patients with PIH also had significantly higher illness severity, as reflected by higher APACHE II (25.3 ± 6.4 vs. 19.8 ± 5.7; t = 6.22, p < 0.001) and SOFA scores (10.4 ± 2.8 vs. 7.8 ± 2.5; t = 6.65, p < 0.001) (Table 1).

**Table 1:** Baseline demographic and clinical characteristics of the study population according to post-intubation hypotension status Continuous variables were compared using the independent-samples t-test, whereas categorical variables were compared using the Pearson Chi-square test. Degrees of freedom for all Chi-square tests were 1. A two-sided p < 0.05 was considered statistically significant. BMI: body mass index; APACHE II: Acute Physiology and Chronic Health Evaluation II; SOFA: Sequential Organ Failure Assessment; SD: standard deviation.

Variable	Post-intubation Hypotension (n = 68)	No Post-intubation Hypotension (n = 166)	Test statistic	p-value
Age (years), mean ±SD	63.8 ± 14.2	56.1 ± 15.1	t = 3.44	0.001
Male sex, n (%)	41 (60.3%)	98 (59.0%)	χ²(1) = 0.03	0.853
BMI (kg/m²), mean ±SD	28.2 ± 4.4	27.1 ± 4.1	t = 1.74	0.083
Hypertension, n (%)	39 (57.4%)	78 (47.0%)	χ²(1) = 2.09	0.149
Diabetes mellitus, n (%)	31 (45.6%)	57 (34.3%)	χ²(1) = 2.58	0.108
Coronary artery disease, n (%)	22 (32.4%)	35 (21.1%)	χ²(1) = 3.24	0.072
Chronic kidney disease, n (%)	18 (26.5%)	23 (13.9%)	χ²(1) = 5.09	0.024
Sepsis, n (%)	35 (51.5%)	46 (27.7%)	χ²(1) = 11.86	<0.001
Trauma, n (%)	12 (17.6%)	42 (25.3%)	χ²(1) = 1.60	0.206
APACHE II score [17], , mean ±SD	25.3 ± 6.4	19.8 ± 5.7	t = 6.22	<0.001
SOFA score [16], , mean ±SD	10.4 ± 2.8	7.8 ± 2.5	t = 6.65	<0.001

Patients who developed PIH had significantly lower pre-intubation systolic blood pressure (101.4 ± 13.8 vs. 118.6 ± 16.5 mmHg; t = −8.16, p < 0.001) and a higher shock index (1.08 ± 0.26 vs. 0.82 ± 0.19; t = 7.47, p < 0.001). Ketamine was used more frequently among patients who developed PIH (n=44, 64.7% vs. n=77, 46.4%; χ² = 6.48, p = 0.012), whereas etomidate was administered more commonly in patients who remained hemodynamically stable (n=24, 35.3% vs. n=89, 53.6%; χ² = 6.48, p = 0.012). Patients with PIH also had a significantly lower first-pass intubation success rate (n=55, 80.9% vs. n=151, 91.0%; χ² = 4.65, p = 0.034), a higher frequency of difficult airway (n=16, 23.5% vs. n=18, 10.8%; χ² = 6.25, p = 0.016), a greater number of intubation attempts (1.41 ± 0.62 vs. 1.19 ± 0.45; t = 2.65, p = 0.005), and a higher incidence of oxygen desaturation during intubation (n=19, 27.9% vs. n=22, 13.3%; χ² = 7.20, p = 0.009) (Table 2).

**Table 2:** Intubation characteristics and hemodynamic parameters Continuous variables were compared using the independent-samples t-test and categorical variables using the Pearson Chi-square test. Degrees of freedom for all Chi-square tests were 1. SBP: systolic blood pressure; SD: standard deviation

Variable	Post-intubation Hypotension (n = 68)	No Post-intubation Hypotension (n = 166)	Test statistic	p-value
Pre-intubation SBP (mmHg), mean ± SD	101.4 ± 13.8	118.6 ± 16.5	t = −8.16	<0.001
Shock Index [15], mean ± SD	1.08 ± 0.26	0.82 ± 0.19	t = 7.47	<0.001
Etomidate use, n (%)	24 (35.3%)	89 (53.6%)	χ²(1) = 6.48	0.012
Ketamine use, n (%)	44 (64.7%)	77 (46.4%)	χ²(1) = 6.48	0.012
First-pass intubation success, n (%)	55 (80.9%)	151 (91.0%)	χ²(1) = 4.65	0.034
Difficult airway, n (%)	16 (23.5%)	18 (10.8%)	χ²(1) = 6.25	0.016
Number of intubation attempts, mean ± SD	1.41 ± 0.62	1.19 ± 0.45	t = 2.65	0.005
Oxygen desaturation (<90%), n (%)	19 (27.9%)	22 (13.3%)	χ²(1) = 7.20	0.009

Patients who developed PIH experienced significantly worse clinical outcomes than those without hypotension. Vasopressor support was required in nearly two-thirds of patients with PIH, occurring in 43 (63.2%) patients compared with 34 (20.5%) patients without PIH (χ² = 39.94, p < 0.001). Patients with PIH also required longer durations of mechanical ventilation (8.9 ± 4.6 vs. 6.2 ± 3.8 days; t = 4.28, p < 0.001), had prolonged ICU stays (10.8 ± 5.2 vs. 7.6 ± 4.3 days; t = 4.49, p < 0.001), and longer hospital stays (15.4 ± 6.7 vs. 11.8 ± 5.9 days; t = 3.86, p < 0.001). Furthermore, AKI occurred more frequently among patients with PIH (21 (30.9%) vs. 23 (13.9%); χ² = 9.16, p = 0.003). ICU mortality (19 (27.9%) vs. 24 (14.5%); χ² = 5.85, p = 0.019) and in-hospital mortality (23 (33.8%) vs. 30 (18.1%); χ² = 6.83, p = 0.011) were also significantly higher in patients who developed PIH (Table 3).

**Table 3:** Clinical Outcomes According to Post-intubation Hypotension Status ICU: intensive care unit; SD: standard deviation. Data are presented as mean ± SD or n (%). Continuous variables were compared using the independent-samples t-test and categorical variables using the Pearson Chi-square test. Degrees of freedom for all Chi-square tests were 1.

Variable	Post-intubation Hypotension (n=68)	No Post-intubation Hypotension (n=166)	Test Statistic	p-value
Vasopressor requirement	43 (63.2%)	34 (20.5%)	χ² = 39.94	<0.001
Mechanical ventilation, days	8.9 ± 4.6	6.2 ± 3.8	t = 4.28	<0.001
ICU stay, days	10.8 ± 5.2	7.6 ± 4.3	t = 4.49	<0.001
Hospital stay, days	15.4 ± 6.7	11.8 ± 5.9	t = 3.86	<0.001
Acute kidney injury	21 (30.9%)	23 (13.9%)	χ² = 9.16	0.003
ICU mortality	19 (27.9%)	24 (14.5%)	χ² = 5.85	0.019
In-hospital mortality	23 (33.8%)	30 (18.1%)	χ² = 6.83	0.011

Multivariable logistic regression identified several factors independently associated with PIH. Increasing age was associated with a 3% increase in the odds of developing PIH for each additional year of age (AOR 1.03, 95% CI 1.01-1.06; Wald χ² = 6.42, p = 0.011). Chronic kidney disease (AOR 2.08, 95% CI 1.01-4.29; Wald χ² = 3.95, p = 0.047), sepsis (AOR 2.64, 95% CI 1.38-5.05; Wald χ² = 8.87, p = 0.003), higher APACHE II score (AOR 1.11, 95% CI 1.04-1.18; Wald χ² = 11.26, p = 0.001), elevated shock index (AOR 3.42, 95% CI 1.74-6.74; Wald χ² = 12.63, p < 0.001), ketamine use compared with etomidate (AOR 1.89, 95% CI 1.01-3.53; Wald χ² = 4.02, p = 0.045), and difficult airway (AOR 2.27, 95% CI 1.07-4.83; Wald χ² = 4.54, p = 0.033) were independently associated with PIH. First-pass intubation success was not independently associated with PIH after multivariable adjustment (AOR 0.54, 95% CI 0.26-1.14; Wald χ² = 2.59, p = 0.107) (Table 4).

**Table 4:** Factors Independently Associated with Post-intubation Hypotension Variables with p <0.20 in univariable analysis or considered clinically relevant were initially considered for multivariable modeling. Pre-intubation SBP was excluded from the final model because of collinearity with Shock Index, which incorporates SBP into its calculation. Shock Index was retained as the preferred composite measure of pre-intubation hemodynamic status. Collinearity diagnostics were performed before final model construction, and no problematic multicollinearity was identified among the variables retained in the final model. β: regression coefficient; SE: standard error; AOR: adjusted odds ratio; CI: confidence interval; APACHE II: Acute Physiology and Chronic Health Evaluation II Regression coefficients (β), standard errors (SE), Wald χ² statistics, adjusted odds ratios (AORs), 95% confidence intervals (CIs), and p values are presented. A two-sided p < 0.05 was considered statistically significant.

Variable	β coefficient	SE	Wald χ²	Adjusted OR	95% CI	p-value
Age (per year increase)	0.030	0.012	6.42	1.03	1.01–1.06	0.011
Chronic kidney disease	0.732	0.369	3.95	2.08	1.01–4.29	0.047
Sepsis	0.971	0.326	8.87	2.64	1.38–5.05	0.003
APACHE II score [17]	0.104	0.031	11.26	1.11	1.04–1.18	0.001
Shock Index [15]	1.230	0.346	12.63	3.42	1.74–6.74	<0.001
Ketamine (vs. etomidate)	0.637	0.318	4.02	1.89	1.01–3.53	0.045
Difficult airway	0.820	0.385	4.54	2.27	1.07–4.83	0.033
First-pass intubation success	−0.616	0.383	2.59	0.54	0.26–1.14	0.107

The multivariable logistic regression model demonstrated acceptable overall fit. The omnibus test of model coefficients was statistically significant (χ² = 58.37, df = 8, p < 0.001), indicating that the model provided a significantly better fit than the intercept-only model. The Hosmer-Lemeshow goodness-of-fit test was non-significant (χ² = 6.18, df = 8, p = 0.627), suggesting adequate calibration. The Cox and Snell R² and Nagelkerke R² values were 0.24 and 0.35, respectively, indicating that the model explained a moderate proportion of the variability in PIH.

PIH was independently associated with several adverse clinical outcomes. After adjustment for clinically relevant baseline covariates, patients with PIH had significantly higher odds of requiring vasopressor support (AOR 4.76, 95% CI 2.53-8.97; Wald χ² = 23.37; p < 0.001), developing acute kidney injury (AOR 2.39, 95% CI 1.19-4.82; Wald χ² = 5.89; p = 0.015), ICU mortality (AOR 2.14, 95% CI 1.08-4.25; Wald χ² = 4.76; p = 0.029), in-hospital mortality (AOR 2.37, 95% CI 1.23-4.58; Wald χ² = 6.60; p = 0.010), and prolonged ICU stay >7 days (AOR 2.81, 95% CI 1.55-5.09; Wald χ² = 11.63; p = 0.001) (Table 5). The specific covariates included in each outcome model are detailed in the footnote to Table 5.

**Table 5:** Association Between Post-intubation Hypotension and Adverse Clinical Outcomes Separate multivariable binary logistic regression models were constructed for each outcome. The vasopressor requirement model was adjusted for age, sex, APACHE II score, sepsis, CKD, and Shock Index. The AKI model was adjusted for age, sex, APACHE II score, CKD, and sepsis. The ICU mortality and in-hospital mortality models were adjusted for age, APACHE II score, CKD, and sepsis. The prolonged ICU stay model was adjusted for age, APACHE II score, CKD, and sepsis. PIH was the primary exposure variable in all models. Variables considered potential mediators of the association between PIH and the outcome were not included in the adjustment models. A two-sided p < 0.05 was considered statistically significant. β: regression coefficient; SE: standard error; AOR: adjusted odds ratio; CI: confidence interval; ICU: intensive care unit; AKI: acute kidney injury; CKD: chronic kidney disease

Outcome	β coefficient	SE	Wald χ²	Adjusted OR	95% CI	p-value
Vasopressor requirement	1.561	0.323	23.37	4.76	2.53–8.97	<0.001
Acute kidney injury	0.871	0.359	5.89	2.39	1.19–4.82	0.015
ICU mortality	0.761	0.349	4.76	2.14	1.08–4.25	0.029
In-hospital mortality	0.863	0.336	6.60	2.37	1.23–4.58	0.010
Prolonged ICU stay (>7 days)	1.033	0.303	11.63	2.81	1.55–5.09	0.001

## Discussion

The present retrospective study evaluated the incidence, factors associated with PIH, and its clinical outcomes among critically ill adults undergoing emergency endotracheal intubation. PIH occurred in 29.1% of patients, consistent with previous reports demonstrating an incidence ranging from 20% to 40% among critically ill adults undergoing emergency airway management [18]. Patients who developed PIH had greater illness severity and experienced significantly worse clinical outcomes, including increased vasopressor requirement, prolonged mechanical ventilation, longer ICU and hospital stays, higher rates of AKI, and increased ICU and in-hospital mortality. Multivariable analysis demonstrated that advanced age, chronic kidney disease, sepsis, higher APACHE II score, elevated shock index, ketamine use, and difficult airway were independently associated with PIH. These findings emphasize the importance of identifying high-risk patients before emergency airway intervention to facilitate appropriate hemodynamic optimization and peri-intubation management.

The observed incidence of PIH in our study is comparable with previously published studies despite differences in study populations, illness severity, airway management practices, and definitions of hypotension. This consistency suggests that PIH remains a frequent complication of emergency airway management even with advances in airway devices and intubation techniques. Given its consistent association with adverse clinical outcomes, PIH continues to represent an important quality indicator of emergency airway management and a potentially preventable contributor to morbidity and mortality [19].

Advanced age and greater illness severity were significantly associated with PIH in the present study. Patients who developed hypotension were older and had significantly higher APACHE II and SOFA scores, indicating more severe physiological derangement before intubation. Older patients generally have reduced cardiovascular reserve and impaired autonomic compensation, making them more susceptible to hemodynamic instability during induction of anesthesia and initiation of positive-pressure ventilation. Similarly, greater illness severity reflects impaired physiological reserve and reduced ability to tolerate the cardiovascular effects of induction agents and mechanical ventilation. These findings are consistent with previous studies reporting advanced age and higher severity scores as important factors associated with peri-intubation hypotension [20].

Sepsis was also independently associated with PIH. More than half of the patients who developed PIH had sepsis as the primary indication for emergency intubation. Septic patients commonly exhibit systemic vasodilation, relative hypovolemia, myocardial dysfunction, and impaired vascular responsiveness, all of which may predispose them to cardiovascular collapse during induction of anesthesia and initiation of mechanical ventilation. Similar observations have been reported in previous studies, highlighting the importance of aggressive hemodynamic optimization, adequate fluid resuscitation, and timely vasopressor support before airway intervention in patients with septic shock [21].

An elevated pre-intubation shock index was another factor independently associated with PIH. Patients with a higher shock index had more than threefold greater odds of developing post-intubation hypotension. The shock index has increasingly been recognized as a simple bedside indicator of occult circulatory compromise and has demonstrated superior predictive ability compared with isolated blood pressure measurements in identifying patients at risk of hemodynamic deterioration. Our findings are consistent with previous studies demonstrating a strong association between elevated shock index and peri-intubation hypotension, supporting its utility as a practical tool for pre-intubation risk stratification [22].

An important finding of this study was the independent association between ketamine use and post-intubation hypotension after adjustment for measured confounding variables. Although ketamine is traditionally considered a hemodynamically stable induction agent because of its sympathomimetic effects, it may produce myocardial depression in patients with prolonged shock or catecholamine depletion. Conversely, etomidate generally preserves myocardial contractility and systemic vascular resistance, contributing to greater hemodynamic stability during induction. However, these findings should be interpreted cautiously. In routine clinical practice, ketamine is often preferentially administered to patients with greater physiological instability or septic shock, introducing the possibility of confounding by indication despite multivariable adjustment. Therefore, the observed association should not be interpreted as evidence that ketamine directly causes post-intubation hypotension. Rather, it likely reflects the complex interaction between patient severity, clinical decision-making, and induction agent selection. Further prospective studies or randomized controlled trials are required to clarify the comparative hemodynamic effects of different induction agents in critically ill patients [23].

Procedural characteristics also appeared to influence the occurrence of PIH. Patients who developed hypotension had lower first-pass intubation success, underwent more intubation attempts, and experienced difficult airway management more frequently than patients without hypotension. Multiple intubation attempts prolong airway manipulation, delay effective oxygenation, and increase physiological stress, thereby increasing the likelihood of peri-intubation complications. Although first-pass success was not independently associated with PIH after multivariable adjustment, difficult airway remained significantly associated with the outcome, emphasizing the importance of meticulous airway planning and experienced operators during emergency intubation. Previous studies have similarly reported associations between difficult airway management, repeated intubation attempts, and peri-intubation hypotension, hypoxemia, and cardiovascular collapse [24].

The adverse clinical outcomes observed among patients with PIH further emphasize its clinical significance. After adjustment for baseline characteristics, PIH remained independently associated with greater vasopressor requirement, AKI, prolonged ICU stay, ICU mortality, and in-hospital mortality. Although causality cannot be established because of the retrospective observational design, these findings support previous evidence suggesting that peri-intubation hemodynamic instability is associated with worse short-term outcomes in critically ill patients. Early recognition of patients at increased risk for PIH, optimization of intravascular volume status, careful selection of induction agents, preparation for vasopressor administration, and strategies aimed at maximizing first-pass intubation success may reduce the incidence and clinical consequences of post-intubation hypotension.

Limitations

This study has several limitations. First, the retrospective observational design precludes establishing causal relationships and relies on the completeness and accuracy of routinely documented medical records. Exclusion of patients with incomplete documentation may have introduced selection bias, as the analyzed cohort may differ systematically from the broader population of emergency intubations. Second, the single-center design and relatively modest sample size may limit generalizability, particularly to ICU, ward, or prehospital intubation settings. Third, residual confounding cannot be excluded, particularly regarding induction-agent selection, difficult airway management, and illness severity, as these factors may be interrelated and influenced by clinician judgment. The modest number of PIH events and data-driven variable selection may also have resulted in model instability; therefore, associations with wider confidence intervals, particularly CKD and ketamine use, should be interpreted cautiously. Fourth, outcome ascertainment may have been influenced by the timing and frequency of blood pressure measurements, and the retrospective records did not consistently provide detailed information regarding measurement modality. The combined PIH definition incorporating absolute thresholds and a relative reduction in SBP may also capture clinically heterogeneous hemodynamic events.

Fifth, the timing of certain clinical variables and outcomes, including APACHE II, SOFA, AKI, and vasopressor use, may not have been uniformly documented with sufficient temporal precision to completely exclude temporal ambiguity. Sixth, important peri-intubation variables, including clinician experience, exact drug dosages, timing of fluid resuscitation, vasopressor administration before intubation, and mechanical ventilator settings immediately after intubation (including positive end-expiratory pressure (PEEP), tidal volume, and respiratory rate), were not consistently available and therefore could not be comprehensively evaluated. These ventilator parameters may influence intrathoracic pressure, venous return, and post-intubation hemodynamics and represent potential sources of residual confounding. Potential overlap between age and APACHE II, and between SBP and Shock Index, was considered during model construction; however, these relationships may still affect interpretation of the adjusted associations. Seventh, duration of mechanical ventilation and ICU/hospital length of stay may be influenced by mortality, which can shorten these measures and introduce competing-event bias; therefore, these outcomes should not be interpreted as unambiguous measures of morbidity. Finally, long-term outcomes, including neurological recovery, functional status, and post-discharge mortality, were not assessed. Prospective multicenter studies with standardized definitions, systematic blood-pressure monitoring, detailed peri-intubation treatment and ventilator data, and appropriate methods for handling competing events are warranted to validate these findings.

## Conclusions

PIH was a common complication among critically ill adults undergoing emergency endotracheal intubation, occurring in nearly one-third of the study population. PIH was associated with increased vasopressor requirement, prolonged mechanical ventilation, longer ICU and hospital stays, AKI, and higher ICU and in-hospital mortality. Advanced age, sepsis, chronic kidney disease, higher APACHE II score, elevated Shock Index, ketamine use, and difficult airway were independently associated with PIH. These findings highlight the importance of early recognition of patients at increased risk and careful peri-intubation hemodynamic management. Given the retrospective single-centre design, the observed associations should not be interpreted as causal, and prospective multicentre studies are warranted to validate these findings and further evaluate strategies for preventing PIH.
